# Pharmacogenetics of pediatric acute lymphoblastic leukemia in Uruguay: adverse events related to induction phase drugs

**DOI:** 10.3389/fphar.2023.1278769

**Published:** 2023-11-17

**Authors:** Gabriela Burgueño-Rodríguez, Yessika Méndez, Natalia Olano, Magdalena Schelotto, Luis Castillo, Ana María Soler, Julio da Luz

**Affiliations:** ^1^ Laboratorio de Genética Molecular Humana, Departamento de Ciencias Biológicas, CENUR Litoral Norte-Sede Salto, Universidad de la República, Salto, Uruguay; ^2^ Red Latinoamericana de Implementación y Validación de Guías Clínicas Farmacogenómicas (RELIVAF-CYTED), Santiago, Chile; ^3^ Servicio Hemato Oncológico Pediátrico (SHOP), Centro Hospitalario Pereira Rossell (CHPR), Montevideo, Uruguay

**Keywords:** acute lymphoblastic leukemia, ALL, toxicity, ancestry, pharmacogenetics, pharmacogenomics

## Abstract

In Uruguay, the pediatric acute lymphoblastic leukemia (ALL) cure rate is 82.2%, similar to those reported in developed countries. However, many patients suffer adverse effects that could be attributed, in part, to genetic variability. This study aims to identify genetic variants related to drugs administered during the induction phase and analyze their contribution to adverse effects, considering individual genetic ancestry. Ten polymorphisms in five genes (*ABCB1*, *CYP3A5*, *CEP72*, *ASNS*, and *GRIA1*) related to prednisone, vincristine, and L-asparaginase were genotyped in 200 patients. Ancestry was determined using 45 ancestry informative markers (AIMs). The sample ancestry was 69.2% European, 20.1% Native American, and 10.7% African, but with high heterogeneity. Mucositis, Cushing syndrome, and neurotoxicity were the only adverse effects linked with genetic variants and ancestry. Mucositis was significantly associated with *ASNS* (rs3832526; 3R/3R vs. 2R carriers; OR: = 6.88 [1.88–25.14], *p* = *0.004*) and *CYP3A5* (non-expressors vs. expressors; OR: 4.55 [1.01–20.15], *p* = *0.049*) genes. Regarding Cushing syndrome, patients with the TA genotype (rs1049674, *ASNS*) had a higher risk of developing Cushing syndrome than those with the TT genotype (OR: 2.60 [1.23–5.51], *p* = *0.012*). Neurotoxicity was significantly associated with *ABCB1* (rs9282564; TC vs. TT; OR: 4.25 [1.47–12.29], *p* = *0.007*). Moreover, patients with <20% Native American ancestry had a lower risk of developing neurotoxicity than those with ≥20% (OR: 0.312 [0.120–0.812], *p* = *0.017*). This study shows the importance of knowing individual genetics to improve the efficacy and safety of acute lymphoblastic leukemia.

## Introduction

In Uruguay, as in the rest of the world, leukemias are the most common pediatric cancer, constituting 30.2% (Kato, 2020; Dufort and Álvarez, 2021). Acute lymphoblastic leukemia (ALL) is the most common childhood leukemia (Maamari et al., 2020). Its incidence rate varies among populations. For instance, it is higher in the Hispanic and European-derived populations of the United States of America (USA) than in the African American and Asian American populations of this country (Fujita et al., 2021). Furthermore, compared with other populations, the incidence rate in Latin American population has increased more rapidly over time (Quiroz et al., 2019; Mejia-Arangure et al., 2021). Overall survival of ALL has remarkably improved, being 10% in the 1960s and reaching up to 90% nowadays (Al-Mahayri et al., 2017). In Uruguay, the ALL survival between 2008 and 2012 was 82.2%, similar to those reported for developed countries such as the United States, some East European countries, Japan, South Korea, and Australia (Castillo et al., 2012; Dufort and Álvarez, 2021).

In Uruguay, the Berlin–Frankfurt–Münster (IC-BFM) protocol is used to treat ALL pediatric patients, who are classified into three risk groups (standard, intermediate, and high), according to the age of diagnosis, white blood cell (WBC) count, presence of translocations, peripheral blood blast number on day +8 (PBB+8), and minimal residual disease (MRD) on days +15 and +33. The overall treatment consists in a 2-year chemotherapy divided into five phases. The first phase, induction to remission, consists of the administration of prednisone (PRED), vincristine (VCR), L-asparaginase (L-ASP), daunorubicin, and methotrexate (MTX). Although these drugs have shown high efficiency, their non-specific action and high administered doses make them potentially toxic (Gervasini and Vagace, 2012). The inter-individual variability in the treatment response can be explained by non-genetic (age, sex, concomitant diseases, and diet) and genetic factors (Arribas and Pérez, 2010). One of the most common adverse events is oral mucositis, occurring in 52%–80% of children treated with chemotherapeutic regimens (Cheng et al., 2008). Although some studies have reported an association between oral mucositis and genetic polymorphisms in different genes such as *ABCB1*, *ABCC2*, *ABCG2*, and *MTHFR* (Tantawy et al., 2010; Bektaş-Kayhan et al., 2012; Liu et al., 2014; Viana Filfo et al., 2021), these studies have not been able to be replicated.

PRED and dexamethasone are two glucocorticoids (GCs) administered in ALL therapy that increase therapeutic efficacy (Kawedia et al., 2011). However, GC administration has been associated with a variety of unwanted adverse effects, such as skin fragility, weight gain, increased infection risk, metabolic and cardiovascular impact, hypertension, hyperglycemia, Cushing syndrome, dyslipidemia, and bone toxicity (Schijvens et al., 2019). ABCB1 protein is a drug efflux pump with broad substrate specificity that can expulse PRED, VCR, MTX, and anthracyclines to the extracellular space (Gregers et al., 2015; Song et al., 2017). Therefore, any variation in the expression or activity levels of this protein could affect the response to the ALL treatment (Fletcher et al., 2010; Song et al., 2017; Mohammad et al., 2018). The relationship between rs2032582 (A>C/T) in the *ABCB1* gene and the response to ALL treatment has been reported. In a study conducted in a Malay, Chinese, and Indian children cohort, the AA genotype was associated with lower event-free survival and a higher risk of relapse (Lu et al., 2014). On the other hand, a study in a Chinese population reported that individuals with CC, CT, and TT genotypes had a higher risk of relapse or death compared with CA, AA, or AT genotypes. However, no relationship was found between this variant and WBC at debut, PBB+8 (Zhai et al., 2012; Gasic et al., 2018), and MRD on days +15 and +33 (Zhai et al., 2012).

VCR is a plant alkaloid that exerts cytotoxic effects by interfering with the microtubule assembly and mitotic spindle formation. This inhibition leads to apoptosis (Jordan and Wilson, 2004; Echebarria Barona, 2016; Al-Mahayri et al., 2017). In the nervous system, VCR disrupts axonal microtubules, causing axonal inflammation in both myelinated and unmyelinated fibers. Consequently, sensory and motor functions are affected, thus influencing the patient’s life quality during or even after treatment (Carozzi et al., 2015; Martin-Guerrero et al., 2019). Several variables could shape the incidence of VCR-induced neurotoxicity: dose, cumulative dose, administration frequency, interaction with other drugs, genetic ancestry, gene variants, and even the methods used to determine this incidence (Sims, 2016; Stock et al., 2017; McClain et al., 2018; Laushke et al., 2019). In the liver, the CYP3A5 enzyme is the main VCR metabolizing enzyme, generating three inactive metabolites (Dennison et al., 2006; Lamba et al., 2012). *CYP3A5 g*enetic variability has been associated with VCR-induced neurotoxicity both *in vitro* and *in vivo* (Aplenc et al., 2003; Egbelakin et al., 2011; Lamba et al., 2012). Homozygous or compound heterozygous individuals for *CYP3A5* *3, *6, and *7 alleles present a higher risk of developing VCR-induced peripheral neuropathy events (Egbelakin et al., 2011). However, some studies did not find an association between the *CYP3A5* genotype and neurotoxicity (Ceppi et al., 2014). Additionally, the *CEP72* gene (which encodes for an essential centrosomal protein in the microtubule construction) contains a polymorphism (rs924607) whose role in VCR-induced neurotoxicity has been discussed (Diouf et al., 2015; Gutiérrez-Camino et al., 2016; Stock et al., 2017). Concerning genetic ancestry, a higher frequency of VCR-induced neuropathies has been reported in European-derived children than in African American and Hispanic children (Renberger et al., 2008; Sims, 2016; McClain et al., 2018).

L-ASP is an ASNase that catalyzes the L-asparagine hydrolysis into L-aspartic acid and ammonia, depriving tumor cells of an essential growth factor (asparagine), resulting in their death (Verma et al., 2007; Shrivastava et al., 2016; Burke and Zalewska-Szewczyk, 2022). Although L-ASP administration has been one of the main contributions to pediatric ALL treatment in the last 50 years, it has also been associated with different adverse effects, among which hypersensitivity to the drug is prominent. Some studies have linked the occurrence of L-ASP allergy with the patient risk group (Chen et al., 2010; Kutszegi et al., 2015; Rajic et al., 2015). Furthermore, there are several investigations about variants in *ASNS* and *GRIA1* genes and their influence on this adverse effect (Akagi et al., 2009; Chen et al., 2010; Pastorczak et al., 2014; Ben Tanfous et al., 2015; Kutszegi et al., 2015; Rajic et al., 2015). Moreover, Wacker et al. (2007) reported a higher frequency of L-ASP allergic reactions in pediatric ALL European-derived populations than in Hispanic or Afro-descendant populations. In agreement, Chen et al. (2010) reported that American–Indian ancestry is associated with a lower risk of developing L-ASP hypersensitivity. However, Kahn et al. (2018) did not find a significant difference in the incidence of L-ASP-associated toxicities between Hispanic and non-Hispanic children.

From a historical and genetic perspective, the Uruguayan population is considered an admixed population with at least three ancestries (Sans et al., 1997; Bonilla et al., 2004; Hidalgo et al., 2005; Bonilla et al., 2015). According to history, the Uruguayan territory prior to the European conquest was occupied by the Charrúas, Minuanes, Arachanes, Chanás, and Guaraníes Native American groups (Cabrera, 1992). The colonization of Uruguay occurred mainly by the Spanish (from Asturias, the Canary Islands, and Galicia) and Portuguese (from Portugal and Brazil) (Pi and Vidart, 1969; Sans et al., 1997; Sans et al., 2021). Due to the slave trade, Uruguay also received African populations of diverse origins, especially of Bantu ethnicity (Isola, 1975). Although recently Uruguay does not have Afro-Uruguayan or Native American isolated communities, the contribution of these populations is observed in the Uruguayans’ genetic background. The European genetic contribution varies between 70% and 80%, whereas the Native American and African contributions vary between 10%–14% and 6%–9.5%, respectively (Sans et al., 2021). This genetic structure is very enthralling for carrying out pharmacogenetics studies.

This investigation aims to identify genetic variants related to the drugs administered during the induction phase of pediatric ALL therapy (PDR, VCR, and L-ASP) and analyze their contribution to response and adverse effects, considering individual genetic ancestry.

## Methodology

The protocol and procedure employed were in accordance with the principles of the Declaration of Helsinki and approved by the *CENUR Litoral Norte, Universidad de la República*, institutional ethics committee. Informed consent was obtained from parents, guardians, and patients, as required (Exp. 311170-001142-19, www.expe.edu.uy).

### Patient samples

This investigation is a retrospective molecular epidemiology study. A total of 200 pediatric patients diagnosed with ALL (between 1 and 19 years old) were analyzed. Patients were treated according to the IC-BFM protocol at the *Servicio Hemato Oncológico Pediátrico—Centro Hospitalario Pereira Rossell (SHOP-CHPR)*, *Montevideo*, *Uruguay*, and recruited between 2010 and 2020. SHOP-CHPR is the national reference center for pediatric ALL and treats children from the entire county. Patients’ clinical and demographic data are detailed in Supplementary Table S1. DNA was extracted from peripheral blood white cells using the salting-out method (Miller et al., 1988).

### Genotyping

Ten polymorphisms in *ABCB1*, *CYP3A5*, *CEP72, ASNS*, and *GRIA1* genes were genotyped by different molecular approaches such as PCR, PCR-RFLP, PCR-HRM, and TaqMan probes (Supplementary Table S2). For validation purposes, all patients carrying a rare variant allele, as well as a similar number of non-carriers, were subjected to Sanger sequencing. For frequent variants, 10% of the samples were selected to be sequenced, considering the genotype proportions. Individual and global ancestry were determined by genotyping 45 ancestry informative markers (AIMs), selected from the SNP panel published by Yaeger et al. (2008). The eligibility criteria were that they were adequately distributed across the genome and that at least one was in each of the autosomes. Moreover, the selection tried to contemplate a similar proportion of AIMs that can distinguish between the three possible pairs of ancestral populations. Nineteen AIMs were analyzed using SNaPshot multiplex (Thermo Fisher Scientific, Waltham, Massachusetts, United States), and 26 AIMs were analyzed by MassARRAY SNP genotyping (Agena Bioscience Inc., San Diego, United States).

### Clinical and paraclinical data

From medical records, several clinical and paraclinical data were obtained: the number of blasts on days +1, +8, +15, and +33 and MRD on days +15 and +33, and relapses or deaths. Moreover, for each of the 33 days of the induction phase, a complete blood count and clinical symptoms such as infection, mucositis, erythema, gastrointestinal disorders, allergies, Cushing syndrome, and hyperglycemia were collected. Furthermore, neurological events and L-ASP allergy were checked throughout the treatment. These data were collected blinded to genotypes. The current investigation focused only on PRED response on day +8, mucositis, Cushing syndrome, L-ASP hypersensitivity, and neurotoxicity. Mucositis and Cushing syndrome were classified into severity grades, according to NIH-NCI Common Terminology Criteria for Adverse Events (CTCAE). Neurological toxicity was defined following the Spanish Society of Medical Oncology criteria (Blasco and Caballero, 2019), according to the presence of any of the following symptoms: progressive confusion, hallucinations, aphasia, speech disturbance, lethargy, drowsiness, seizures, ataxia, dysmetria, dysarthria, nystagmus, facial droop, rapid eye movement, loss of sensation in extremities, and paresthesia.

### Statistical analysis

Genotypic and allelic frequencies were calculated by gene counting. The Hardy–Weinberg equilibrium (HWE) was estimated using Fisher’s exact test, and genotypic frequencies were compared to 1000 Genomes Project Consortium populations using the population differentiation test in Arlequin software v3.5.2 (Excoffier and Lischer, 2010). Moreover, the F-statistic *FIS* was calculated, according to Hedrick (2011). Individual and global genetic ancestry were calculated using STRUCTURE 2.3.4 software (Pritchard et al., 2000) using the following parameters: K = 3 (European, Amerindian, and African), 100,000 iterations for the burn-in period, and 1,000,000 additional iterations. The parental populations included 42 Europeans (Coriell’s North American panel), 37 West Africans (non-admixed Africans living in London, United Kingdom, and South Carolina, United States), and 30 Native Americans (15 Mayans and 15 Nahuas), who were genotyped on an Affymetrix 100 K SNP chip (data were kindly provided by Dr. Fejerman, University of California, San Francisco).

The relationships between ancestry and PRED response, and ancestry and toxicities were analyzed using the Mann–Whitney (MW) test, considering each ancestry separately. In some analyses, these components were dichotomized using a cut-off value based on their mean. The PRED response was measured as PBB+8 and dichotomized into two groups: <1000 and ≥1000 blasts. Toxicities were analyzed as presence/absence. Regarding *CYP3A5*, patients were classified as expressors or non-expressors. The formers carry at least one functional allele (*1), and the latter are either homozygous for one variant or compound heterozygous. The analysis between categorical variables was performed using the chi-squared test. When the chi-squared test showed significant differences, odd ratio (OR) risk analyses were performed. Additionally, the relationship between the number of events of each toxicity and genetic variants was analyzed using the MW test. Finally, classification and regression trees were performed using the Chi-squared Automatic Interaction Detector (CHAID) algorithm to analyze the toxicities, including genetic variants and ancestry. Statistical analyses were carried out using SPSS 22.0 software (IBM Corp Resealsed, 2013) with a significance *p*-value of 0.05.

## Results

Of the 200 patients, only 184 had medical records available. Furthermore, ancestry was successfully determined in 197 samples. Since not all patients were genotyped for all variants (due to the lack of DNA), the number of patients included in each analysis may differ. Genotypic, allelic frequencies, HWE, and *FIS* for the 10 variants are detailed in Table 1. With the exception of rs1049674 (*ASNS*), the others were found in HWE. For most polymorphisms, the patient sample differs from the African populations, followed by the Asians, and, to a lesser extent, from the Europeans and the Latin American populations (Supplementary Table S3).

**TABLE 1:** Genotype and allele frequencies.

Gene	Variant	Genotype	N	Frequency	Allele	Frequency	HWE *p*-value*	*FIS*
***ABCB1***	rs2032582	CC	56	0.354	C	0.611	0.684	−0.070
	(N = 158)	CA	77	0.487	A	0.370		
		CT	4	0.025	T	0.019		
		AA	19	0.120				
		AT	2	0.013				
		TT	0	0.000				
	rs9282564	TT	155	0.886	T	0.943	1.000	−0.058
	(N = 175)	TC	20	0.114	C	0.057		
		CC	0	0.000				
***CYP3A5***	rs776746 (*3)	*1/*1	3	0.019	*1	0.108	0.521	0.069
	rs10264272 (*6)	*1/*3	28	0.177	*3	0.886		
	rs41303343 (*7)	*3/*3	125	0.791	*6	0.003		
	(N = 158)	*3/*6	1	0.006	*7	0.003		
		*3/*7	1	0.006				
***CEP72***	rs924607	CC	61	0.377	C	0.593	0.192	0.108
	(N = 162)	CT	70	0.432	T	0.407		
		TT	31	0.191				
***ASNS***	rs3832526	2R2R	101	0.605	2R	0.766	0.200	0.099
	(N = 167)	2R3R	54	0.323	3R	0.234		
		3R3R	12	0.072				
	rs1049674	TT	99	0.623	T	0.811	***0.001***	−0.229
	(N = 159)	TA	60	0.377	A	0.189		
		AA	0	0.000				
***GRIA1***	rs4958351	GG	80	0.516	G	0.710	0.440	0.064
	(N = 155)	GA	60	0.387	A	0.290		
		AA	15	0.097				
	rs11951398	CC	142	0.899	C	0.949	1.000	−0.050
	(N = 158)	CT	16	0.101	T	0.051		
		TT	0	0.000				

N, number of patients; HWE, Hardy–Weinberg equilibrium; *, Fisher’s exact test; *FIS*, inbreeding coefficient; CYP3A5*1, reference or native allele.

Bold-italic values represents the significative *p*- values

The study population showed a tri-hybrid structure, with the European ancestral proportion being the main one (69.2% ± 14.0%), followed by the Native American (20.1% ± 12.3%) and the African (10.7% ± 7.4%). Regarding individual ancestry, the distribution of the three ancestral components presented high heterogeneity (Supplementary Figure S1).

Concerning ancestry and PRED response, patients with <1000 PBB+8 had a higher proportion of Native American ancestry (*p* = *0.034*; Supplementary Figure S2). It was not possible to perform an OR analysis with a Native American cut-off value of 20% since all patients with more than 20% had <1000 PBB+8. None of the two variants analyzed in *ABCB1* (rs2032582 and rs9282564) showed statistically significant differences concerning PRED response.

**FIGURE 1:**
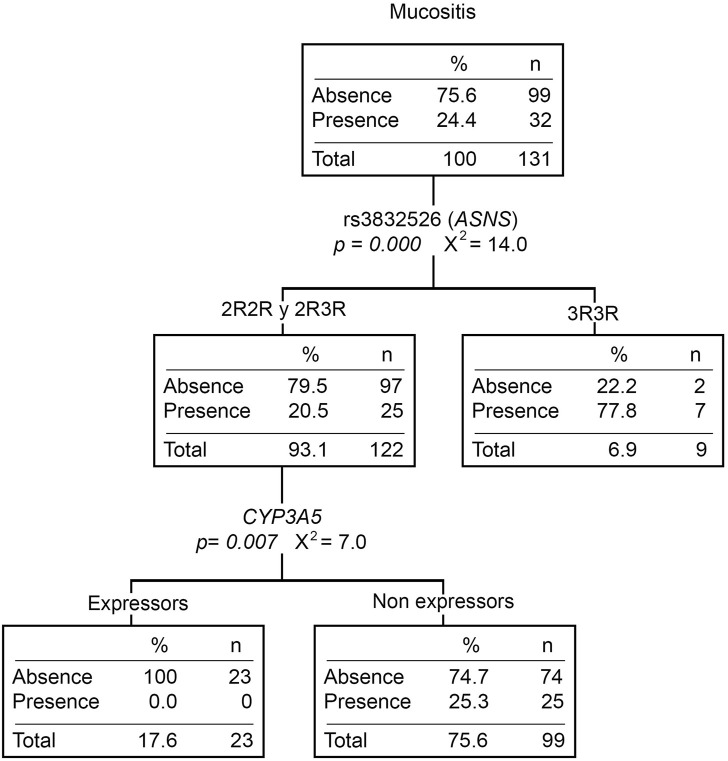
Mucositis classification tree. The presence/absence of mucositis was considered the dependent variable. *ABCB1* and *ASNS* genotypes, the *CYP3A5* phenotype (expressors and non-expressors), and Native American ancestry (greater or less than 20%) were considered independent variables. n, number of patients; *p*, *p*-value; X^2^, chi-squared coefficient.

The relationship between gene variants and toxicities is shown in Supplementary Table S4. Twenty-four percent (N = 44) of the patients developed mucositis during the induction phase (32% were in grades two or three). Patients who do not express *CYP3A5*, as well as those with the 3R/3R genotype (rs3832526, *ASNS*), had a higher risk of developing mucositis (OR = 4.55 [1.01–20.15], *p* = *0.049* and OR = 6.88 [1.88–25.14], *p* = *0.004*) (Supplementary Table S5). Moreover, the *CYP3A5* non-expressors or the 3R/3R patients present a higher number of mucositis events (*p* = *0.034* and *0.001*, respectively). Likewise, the mucositis classification tree shows both *CYP3A5* and rs3832526 as the only variables that explain the presence of this toxicity (Figure 1). Ancestry was not associated with this toxicity either (Supplementary Table S6).

**FIGURE 2:**
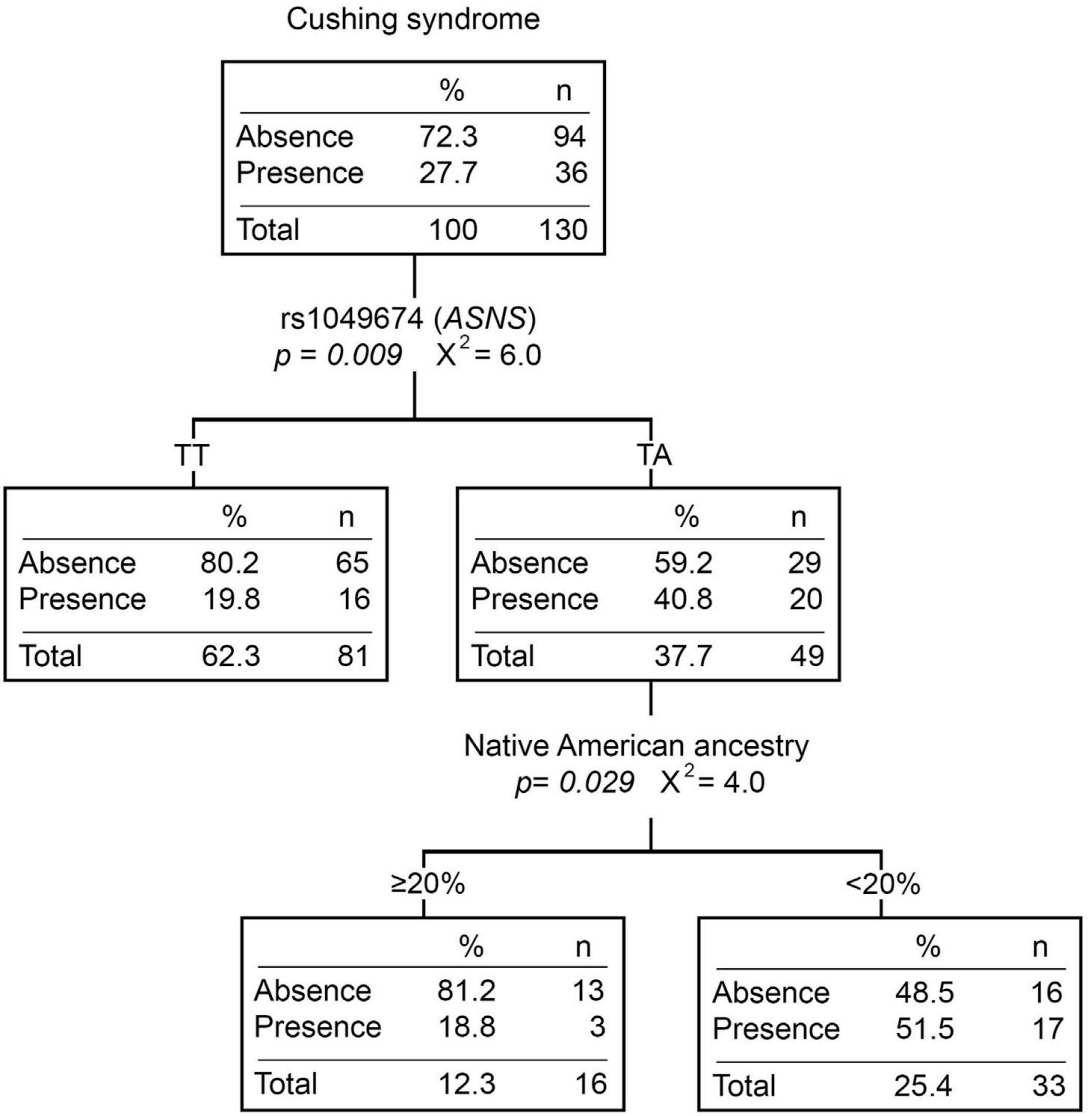
Cushing syndrome classification tree. The presence/absence of Cushing syndrome was considered the dependent variable. *ABCB1* and *ASNS* genotypes, and Native American ancestry (greater or less than 20%) were considered independent variables. n, number of patients; *p, p*-value; X^2^, chi-square coefficient.

Twenty-eight percent (N = 51) of the patients presented at least one Cushing event during the induction phase. Patients with the TA genotype for rs1049674 (*ASNS*) had a higher risk of developing Cushing syndrome compared to those with the TT genotype (OR = 2.60 [1.23–5.51], *p* = *0.012*) (Supplementary Table S5). Moreover, patients with the TA genotype presented more Cushing events than those with the TT genotype (*p* = *0.005*). Even though Cushing syndrome did not show statistically significant differences with any of the ancestries (Supplementary Table S6), the classification tree divided the TA genotype patients by the Native American ancestry. The percentage of patients with Cushing syndrome was higher in those with <20% Native American ancestry (Figure 2).

Forty percent of the patients (N = 74) developed L-ASP allergy during treatment (6.7% during the induction phase). Neither *ASNS* and *GRIA1* variants nor ancestry was statistically significantly associated with L-ASP hypersensitivity (Supplementary Tables S4, S6).

**FIGURE 3:**
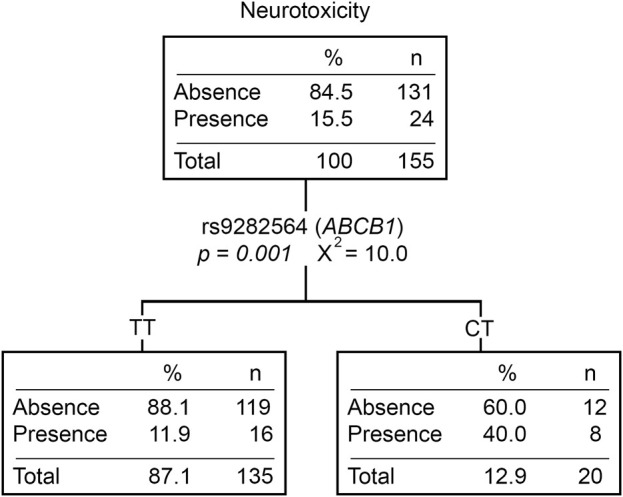
Neurotoxicity classification tree. The presence/absence of neurotoxicity was considered the dependent variable. *ABCB1*, *CEP72*, *ASNS* genotypes, *CYP3A5* phenotype (expressors and non-expressors), and Native American ancestry (greater or less than 20%) were considered independent variables. n, number of patients; *P*, *p*-value; X^2^, chi-squared coefficient.

Twenty-nine patients (15.7%) experienced at least one neurological event during treatment, mostly in post-induction phases (83%). Patients with the CT (rs9282564, *ABCB1*) genotype had a higher risk of developing neurological toxicity than those with the TT genotype (OR = 4.25 [1.47–12.29], *p* = *0.007*) (Supplementary Table S5). Moreover, patients with neurotoxicity had lower Native American ancestry (*p* = *0.002*). Those patients with ≥20% Native American ancestry had a lower risk of developing this toxicity compared to those with <20% (OR = 0.31 [0.12–0.81], *p* = *0.017*). When analyzing neurotoxicity with the Native American ancestry and genotypic variants, the CT genotype of rs9282564 was the only explanatory variable (Figure 3).

## Discussion

Only rs1049674 (*ASNS*) was not found in HWE, showing an excess of heterozygotes (Table 1). This equilibrium deviation could be due to a genotyping error, the existence of evolutionary factors, population substructure, or admixture linkage disequilibrium (ALD), as reported by Bonilla et al. (2015). Since this polymorphism was also not found in HWE in a control sample (data not shown) and was validated by Sanger sequencing, a genotyping error was discarded as an explanation. As rs1049674 was in linkage equilibrium with most of the polymorphisms analyzed (data not shown), the rest of the polymorphisms were in HWE, and the *FIS* values were near zero (either positive or negative), a population substructure or ALD are not the most plausible explanations. This equilibrium deviation needs to be further addressed.

The ancestry heterogeneity observed and the global ancestry agree with genetic and sociodemographic data previously reported for Uruguay (Instituto Nacional de Estadística, 2011; Sans et al., 2021). However, the Native American and African ancestry are slightly higher than those previously published for Uruguay (Sans et al., 1997; Bonilla et al., 2004; Bonilla et al., 2015; Sans et al., 2021). This difference could be attributable to the fact that most of the patients belong to the public healthcare system. According to Bonilla et al. (2015) and Sans et al. (2021), Native American and African ancestries are slightly higher in the public healthcare system population, showing heterogeneity according to the socioeconomic level. Additionally, the association between the Native American ancestry and ALL risk (Walsh et al., 2013) could also explain the observed higher proportion of this ancestry.

Furthermore, patients with <1000 PBB+8 had a significantly higher Native American ancestry than those with ≥1000 (Supplementary Figure S2), suggesting a better PRED response. This result does not agree with other studies that associate a worse treatment response with the Native American ancestry (Bhatia et al., 2002; Kadan-Lottick et al., 2003; Bhatia, 2011; Yang et al., 2012; Quiroz et al., 2019; Lee et al., 2022). Therefore, there could be other genetic and non-genetic factors associated with the Native American component that should be studied.

Despite oral mucositis being one of the most frequent side effects of antineoplastic therapy, few studies analyze possible genetic risk factors. In this study, *CYP3A5* non-expressors and patients with the 3R3R genotype (rs3832526, *ASNS*) showed a higher risk of developing mucositis and a higher number of mucositis events than those who express *CYP3A5* and carry the 2R allele, respectively. The CYP3A5 enzyme converts VCR into inactive metabolites. A deficiency of this enzyme activity could lead to VCR accumulation, which could induce the development of toxicities (López-López et al., 2016). On the other hand, some studies associate mucositis with L-ASP administration, but none of them investigate the role of genetic variants (Müller et al., 2000; Ortiz et al., 2013). The 3R3R genotype has been associated with a higher risk of developing L-ASP hypersensitivity (Ben Tanfous et al., 2015). Although hypersensitivity generally manifests as anaphylaxis, edema, urticaria, and erythema, among others (Fonseca et al., 2021), we cannot rule out mucositis as a possible manifestation.

Moreover, Cushing syndrome was associated with rs1049674 (*ASNS*). Patients with the TA genotype for rs1049674 had a higher risk of developing Cushing syndrome and more events than those with the TT genotype. Although the impact of this variant is unknown, changes in *ASNS* expression could cause greater sensitivity to L-ASP and lead to the development of Cushing’s syndrome. Moreover, the percentage of TA patients that developed Cushing syndrome was higher among those with <20% Native American ancestry than those with ≥20% (Figure 2). Although we cannot rule out that the Native American ancestry protective effect is due to parental population frequencies of rs1049674, when a multidimensional scaling (MDS) is constructed from genetic distances (F_ST_), the subpopulation without Cushing syndrome is clustered with Latin American populations and split from the one with Cushing syndrome (Supplementary Figure S3A).

Similar to previous reports, 40.2% of the patients developed L-ASP allergy during treatment, especially in the post-induction phases. Neither *ASNS-* or *GRIA1-* analyzed variants nor ancestry were significantly associated with L-ASP allergy. Since there are contradictory results on the role of *ASNS* and *GRIA1* variants in this toxicity (Zalewska-Szewczyk et al., 2007; Chen et al., 2010; Pieters et al., 2011; Ben Tanfous et al., 2015; Kutszegi et al., 2015; Rajic et al., 2015; Youssef et al., 2021), the lack of association was not a surprise. Regarding ancestry, the difference between our result and that reported by Chen et al. (2010) could be explained by different admixture proportions or different Native American ancestral populations.

Given that 83% of the neurotoxicity observed was in post-induction phases, it cannot be affirmed that this toxicity is only due to VCR administration. Other drugs, such as MTX, could also influence this toxicity development (Vezmar et al., 2003; Bhojwani et al., 2014). Those patients with the CT genotype for rs9282564 (*ABCB1*) showed a higher risk of developing this adverse effect than those with the TT genotype. Until now, this variant has not been associated with adverse effects derived from ALL therapy. Since VCR and MTX are transported outside the cell by *ABCB1* (Whirl-Carrillo et al., 2012), variants that modify the activity of this transporter could cause various toxicities, such as the neurological toxicity. On the other hand, those patients with neurological toxicity had a significantly lower Native American ancestry than those who did not. As with PRED and Cushing syndrome, a relationship between having a higher Native American ancestry and a lower risk of developing toxicity is depicted. Although some investigations report an association between neurotoxicity and the ancestry (Renbarger et al., 2008; Sims, 2016; McClain et al., 2018), they are mostly based on the high frequency of *CYP3A5* expressors in Afro-descendent populations.

Since the Native American ancestry and rs9282564 (*ABCB1*) were not associated (data not shown), these two variables would independently explain the presence of neurotoxicity. However, the classification and regression tree for neurotoxicity showed that the only explanatory variable was the rs9282564 genotype (Figure 3). It is possible that the loss of Native American ancestry association is due to the fact that not all the patients had the entire information for this analysis (toxicity, ancestry, and genetic variants). Although the Native American ancestry does not reach the desired significance, we cannot discard a possible effect of it on neurological toxicity. Moreover, MDS does not allow ruling out the implications of the Native American ancestry. Since rs9282564 does not properly discriminate between the 1000 genome populations, it is not possible to assign either of the two subpopulations (neuro and non-neuro) to a particular genetic background (Supplementary Figure S3B). It would be desirable to expand this analysis to determine the importance of Native American ancestry in neurotoxicity.

The associations hitherto observed between the ancestry and treatment response/toxicities show an apparent protection of the Native American ancestry. This could be due to several factors: a) the homogeneity in health access of these patients could reveal genetic factors that might not be observed in other studies, b) the Uruguayan Native American ancestry may not be the same reported for other admixed populations, and c) micro-evolutionary factors such as genetic drift or bottlenecks that have varied the frequency of variants within the Native American ancestry.

To the best of our knowledge, this is the first report that investigates side effects caused by drugs administered during the induction phase in Latin America. Even though 200 may be considered a small number, Uruguay has approximately 20 new pediatric patients with ALL per year. Hence, analyzing a 200-individual sample represents more than 10 years of ALL patients in our country. Moreover, the treatment response homogeneity among patients, regardless of patients’ socioeconomic levels, is another strength of this investigation. As for the weaknesses, although estimating an ancestry with 45 AIMs is a valid approach, some works recommend a minimum use of 50 markers (Russo et al., 2016). Moreover, having genomic data from ancestral populations more representative of the Uruguayan population would allow the ancestry estimation to be more precise. To conclude, this investigation highlights the importance of studying Latin American populations due to their differences with ancestral populations and their high heterogeneity.

## Data Availability

The raw data supporting the conclusion of this article will be made available by the authors, without undue reservation.
