# Supplementary material for: Pharmacogenetics of pediatric acute lymphoblastic leukemia in Uruguay: adverse events related to induction phase drugs
Source: Front Pharmacol. 2023 Nov 17;14:1278769. doi: 10.3389/fphar.2023.1278769 (PMC10690766; doi:10.3389/fphar.2023.1278769)
Supplement: Supplementary file 1 [file DataSheet2.PDF]

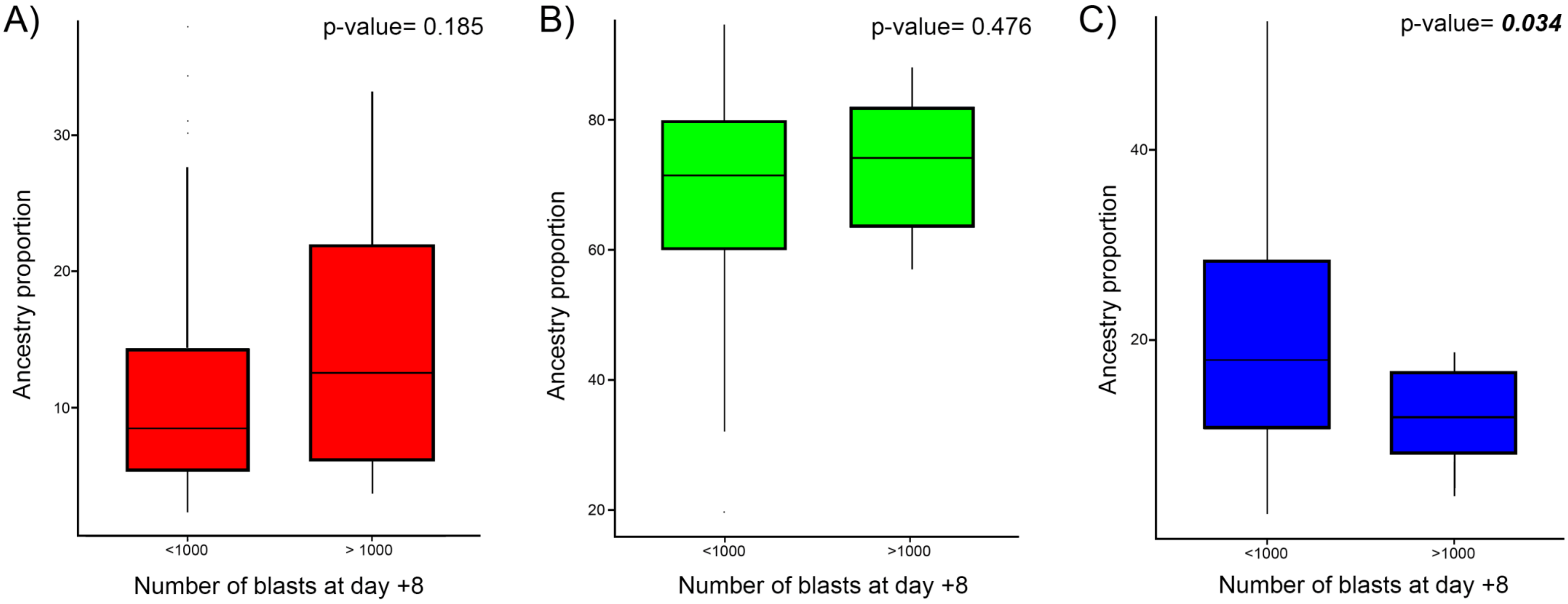

Supplementary Figure 2. Relationship between ancestry and the number of blasts at day +8. Each graph shows the relationship between an ancestral genetic component and the number of blasts at day +8, according to the cut-off value of 1000. The p-value for the Mann-Whitney test is indicated on the upper right edge of each graph. A) African (red). B) European (green). C) Native American (blue)
