# Supplementary material for: Pharmacogenetics of pediatric acute lymphoblastic leukemia in Uruguay: adverse events related to induction phase drugs
Source: Front Pharmacol. 2023 Nov 17;14:1278769. doi: 10.3389/fphar.2023.1278769 (PMC10690766; doi:10.3389/fphar.2023.1278769)
Supplement: Supplementary file 2 [file Table1.DOCX]

# Supplementary Table 1. Patients’ clinical and demographic data

| **Sex** | **N (%)** |  | **Risk group** | **N(%)** |
| --- | --- | --- | --- | --- |
| Female | 87 (43.5) |  | Standard | 35 (17.5) |
| Male | 113 (56.5) |  | Intermediate | 131 (65.5) |
| **Age at diagnosis** |  |  | High | 34 (17.0) |
| 1 - 5 years | 115 (57.5) |  | **CNS** |  |
| 6 - 15 years | 81 (40.5) |  | 1 | 172 (86.0) |
| > 15 years | 4 (2.0) |  | 2 | 15 (7.5) |
| **Immunophenotype** |  |  | 3 | 13 (6.5) |
| B | 184 (92.0) |  | **Relapse (< 5 years)** |  |
| T | 15 (7.5) |  | Yes | 63 (31.5) |
| B & T | 1 (0.5) |  | No | 137 (68.5) |
| CNS: Central Nervous System | | | | |
