# Supplementary material for: Pharmacogenetics of pediatric acute lymphoblastic leukemia in Uruguay: adverse events related to induction phase drugs
Source: Front Pharmacol. 2023 Nov 17;14:1278769. doi: 10.3389/fphar.2023.1278769 (PMC10690766; doi:10.3389/fphar.2023.1278769)
Supplement: Supplementary file 3 [file DataSheet3.PDF]

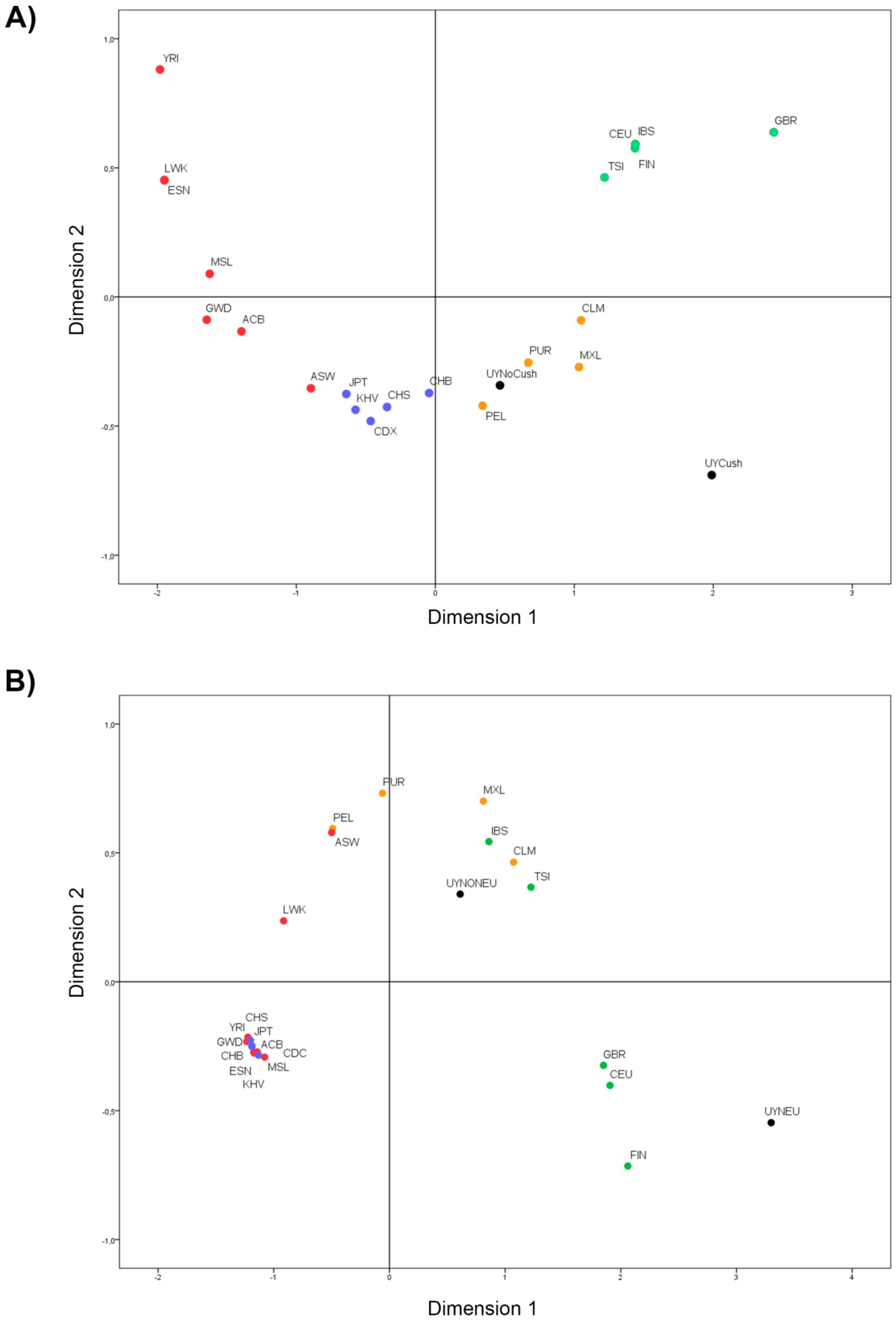

Supplementary Figure 3. **Multidimensional scaling (MDS) based on pairwise FST genetic distances calculated from A) rs1049674 (*ASNS*) and B) rs9282564 (*ABCB1*) genotypes.** A) Stress: 0.03189; RSQ: 0.99618. UYCush & UYNoCush: Uruguayan populations (black). B) Stress: 0.08066; RSQ: 0.98368. UYNeu & UYNoNeu: Uruguayan populations (black). Red: African populations. YRI: Yoruba in Ibadan, Nigeria. ACB: African Caribbeans in Barbados. GWD: Gambian in Western Divisions in the Gambia. MSL: Mende in Sierra Leone. ESN: Esan in Nigeria. LWK: Luhya in Webuye, Kenya. ASW: African Ancestry in Southwest United States. Green: European populations. IBS: Iberian Population in Spain. TSI: Toscani in Italia. CEU: Utah Residents (CEPH) with Northern and Western European Ancestry. FIN: Finnish in Finland. GBR: British in England and Scotland. Blue: East Asian populations. CDX: Chinese Dai in Xishuanagbanna, China. CHB: Han Chinese in Bejing, China. CHS: Southern Han Chinese. JPT: Japanese in Tokyo, Japan. KHV: Kinh in Ho Chi Minh City, Vietnam. Orange: Admixed Latin-American populations. PEL: Peruvians from Lima, Peru. PUR: Puerto Ricans from Puerto Rico. CLM: Colombians from Medellin, Colombia. MXL: Mexican Ancestry from Los Angeles USA. (1000genomes, 2015).
