# Supplementary material for: Pharmacogenetics of pediatric acute lymphoblastic leukemia in Uruguay: adverse events related to induction phase drugs
Source: Front Pharmacol. 2023 Nov 17;14:1278769. doi: 10.3389/fphar.2023.1278769 (PMC10690766; doi:10.3389/fphar.2023.1278769)
Supplement: Supplementary file 4 [file Table2.DOCX]

# Supplementary Table 2. Genes and polymorphisms

| **Gene** | **Polimorphism** | **Methodology** | **Details** |
| --- | --- | --- | --- |
| ***ABCB1*** | rs2032582 | PCR-RFLP (RsaI & BseYI) | Fw: 5’-TCAGCATTCTGAAGTCATGGAA-3’ ^*^ |
|  |  |  | Rv: 5’-TTAGAGCATAGTAAGCAGTAGGGAGT-3’ ^*^ |
|  |  |  | 94°C (5m), 30x [94°C (30s), 60°C (30s),72°C (30s)], 72°C (3m) |
|  | rs9282564 | Taqman probes | Assay ID: C__2614970_10 |
| ***CEP72*** | rs924607 | PCR-RFLP (Hpy188III) | Fw: 5′-GCTTCTGAAGAGCCAGGATG -3′ ^**^ |
|  |  |  | Rv: 5′-TGGCTGTCTCCACTGACAAC-3' ^**^ |
|  |  |  | 95°C (5m), 30x [95°C (20s), 58°C (30s),72°C (30s)], 72°C (5m) |
| ***CYP3A5*** | rs776746 | PCR-Sequencing | Fw: 5’-GTTGTACGACACACAGCAACCT-3’^***^ |
|  |  |  | Rv: 5’-TACCACCCAGCTTAAGCAATGC-3’^***^ |
|  |  |  | 95°C (5m), 30x [95°C (20s), 61°C (30s),72°C (20s)], 72°C (2m) |
|  | rs10264272 | PCR-HRM | Chambliss et al., 2017 |
|  | rs41303343 | PCR-HRM | Chambliss et al., 2017 |
| ***ASNS*** | rs3832526 | PCR | Fw: 5’-ATCCTCCACCCCTTCCTTC-3’^****^ |
|  |  |  | Rv: 5’-ATCACCCTGACCTGCTTACG-3’^****^ |
|  |  |  | 95°C (5m), 30x [95°C (20s), 57°C (30s),72°C (45s)], 72°C (5m) |
|  | rs1049674 | Taqman probes | Assay ID: C__11710748_10 |
| ***GRIA1*** | rs4958351 | PCR-HRM | Fw: 5’-ACACACCTATGTTGGTCCTC-3’ |
|  |  |  | Rv: 5’-GATAGCAGAAGCTATCCCTTAGC-3’ |
|  |  |  | 95°C (5m), 40x [95°C (10s), 61°C (10s),72°C (10s)] |
|  | rs11951398 | PCR-RFLP (BsaAI) | Fw: 5’-AGCATGAGGACGTTGTATTCC-3’ |
|  |  |  | Rv: 5’-AGCCTGGGCTTCTCTCTATAA-3’ |
|  |  |  | 95°C (5m), 30x [95°C (30s), 58°C (30s),72°C (30s)], 72°C (5m) |
| RFLP enzymes are indicated between brackets. *: Yan et al. (2017). **: Gutierrez-Camino et al. (2016). ***: Chambliss et al. (2017). ****: Akagi et al. (2009). | | | |
