# Supplementary material for: Pharmacogenetics of pediatric acute lymphoblastic leukemia in Uruguay: adverse events related to induction phase drugs
Source: Front Pharmacol. 2023 Nov 17;14:1278769. doi: 10.3389/fphar.2023.1278769 (PMC10690766; doi:10.3389/fphar.2023.1278769)
Supplement: Supplementary file 5 [file DataSheet1.PDF]

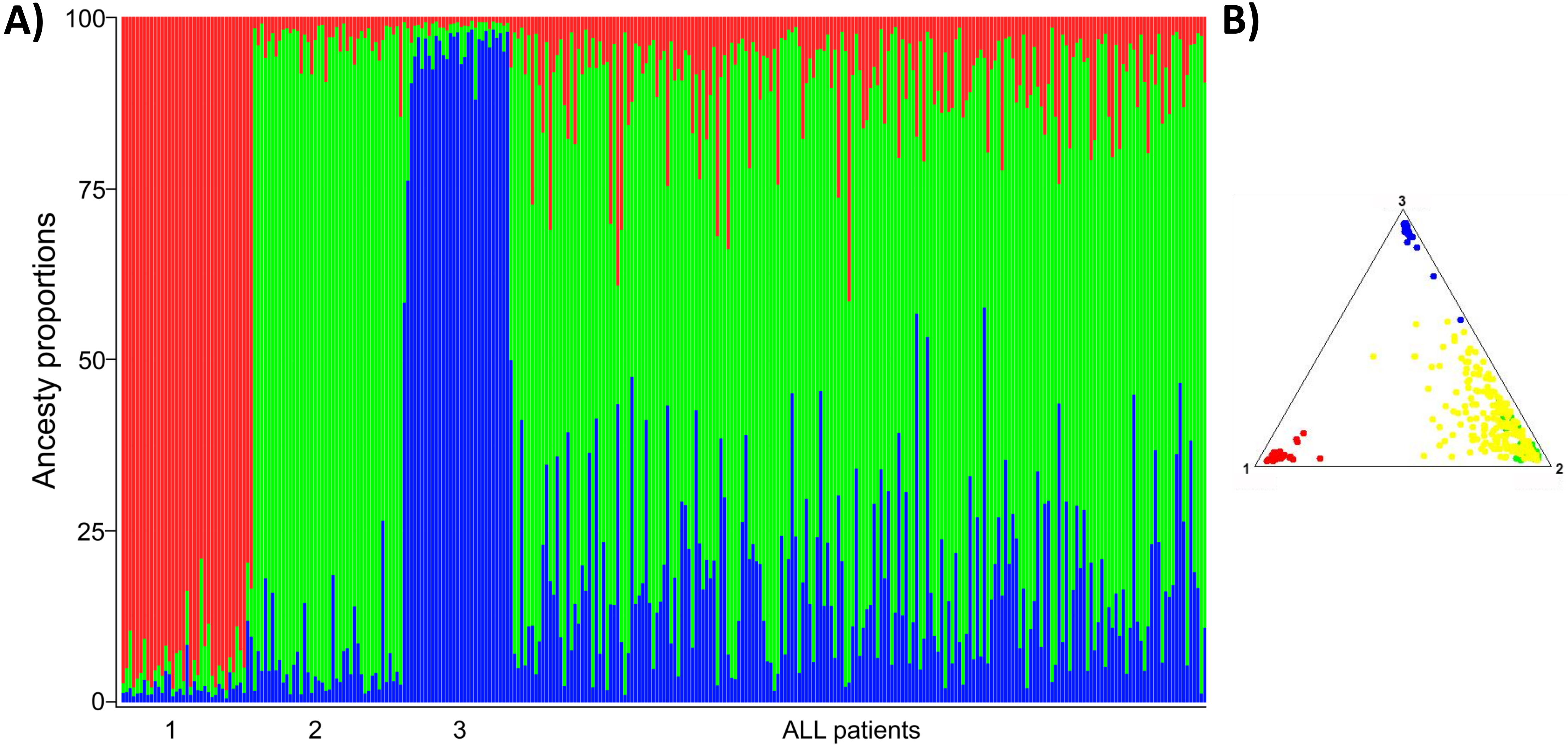

Supplementary Figure 1. Individual ancestry. A) Ancestry bar plot. Each bar represents an individual. **B)** Triangle plot showing the clustering of 197 ALL patients assuming three ancestral populations (k = 3). The percentage of each ancestral component is shown: 1. Africans (37 non-admixed West Africans living in London, United Kingdom, and South Carolina, United States) (red). 2. European (42 individuals from the Coriell's North American panel) (green). 3. Native Americans (15 Mayans and 15 Nahuas) (blue). ALL patients (yellow).
