# Supplementary material for: Pharmacogenetics of pediatric acute lymphoblastic leukemia in Uruguay: adverse events related to induction phase drugs
Source: Front Pharmacol. 2023 Nov 17;14:1278769. doi: 10.3389/fphar.2023.1278769 (PMC10690766; doi:10.3389/fphar.2023.1278769)
Supplement: Supplementary file 6 [file Table5.DOCX]

# Supplementary Table 5: Genetic variants and toxicities risk

| **Toxicity** | **Gene** | **Variant** | **Genotype** | **Absence** | **Presence** | **OR [95%CI]** |
| --- | --- | --- | --- | --- | --- | --- |
|  |  |  |  |  |  | **p-value** |
| Mucositis | ***CYP3A5*** | rs776746 (*3) | expressors^1^ non-expressors^2^ | 25 86 | 2  31 | 4,55 [1,01-20,15] ***0,049*** |
|  |  | rs10264272 (*6) |  |  |  |  |
|  |  | rs41303343 (*7) |  |  |  |  |
|  |  |  | N.A. | 29 | 11 | --- |
|  | ***ASNS*** | rs3832526 | 2R2R / 2R3R | 110 | 28 | 6,88 [1,88-25,14] |
|  |  |  | 3R3R | 4 | 7 | ***0,004*** |
|  |  |  | N.A. | 26 | 9 | --- |
| Cushing | ***ASNS*** | rs1049674 | TT | 73 | 19 | 2,60 [1,23-5,51] |
|  |  |  | TA | 31 | 21 | ***0,012*** |
|  |  |  | N.A. | 29 | 11 | --- |
| Neurotoxicity | ***ABCB1*** | rs9282564 | TT | 124 | 17 | 4,25 [1,47-12,29] |
|  |  |  | TC | 12 | 7 | ***0,007*** |
|  |  |  | N.A. | 19 | 5 | --- |
| OR: odd ratio. CI: Confidence interval. ^1^: *1/*1 and *1/*3. ^2^: *3/*3, *3/*6 and *3/*7. N.A.: Not available | | | | | | |
