# Supplementary material for: Pharmacogenetics of pediatric acute lymphoblastic leukemia in Uruguay: adverse events related to induction phase drugs
Source: Front Pharmacol. 2023 Nov 17;14:1278769. doi: 10.3389/fphar.2023.1278769 (PMC10690766; doi:10.3389/fphar.2023.1278769)
Supplement: Supplementary file 7 [file Table3.DOCX]

# Supplementary Table 3. Differenciation test with 1000 Genome Populations

| **Gene** | **Variant** | **MAF** | *p-value** | | | | | | | | | | | | | | | | | | | | | |
| --- | --- | --- | --- | --- | --- | --- | --- | --- | --- | --- | --- | --- | --- | --- | --- | --- | --- | --- | --- | --- | --- | --- | --- | --- |
|  |  |  | IBS | TSI | CEU | FIN | GBR | ACB | ASW | ESN | GWD | YRI | LWK | MSL | CDX | CHB | CHS | JPT | KHV | CLM | MXL | PEL | PUR |  |
| ***ABCB1*** | rs2032582 | A | 0.977 | 0.697 | 0.672 | 0.399 | 0.706 | ***0.000*** | ***0.000*** | ***0.000*** | ***0.000*** | ***0.000*** | ***0.000*** | ***0.000*** | ***0.000*** | ***0.000*** | ***0.000*** | ***0.000*** | ***0.001*** | 0.182 | 0.480 | ***0.000*** | 0.946 |  |
|  | rs9282564 | C | 0.257 | 0.848 | 0.086 | ***0.013*** | 0.139 | ***0.000*** | ***0.016*** | ***0.000*** | ***0.000*** | ***0.000*** | ***0.001*** | ***0.000*** | ***0.000*** | ***0.000*** | ***0.000*** | ***0.000*** | ***0.000*** | 0.333 | 0.483 | ***0.015*** | 0.082 |  |
| ***CYP3A5*** | rs776746 | T | 0.436 | ***0.040*** | ***0.010*** | 0.118 | 0.113 | ***0.000*** | ***0.000*** | ***0.000*** | ***0.000*** | ***0.000*** | ***0.000*** | ***0.000*** | ***0.000*** | ***0.000*** | ***0.000*** | ***0.000*** | ***0.000*** | 0.073 | ***0.003*** | 0.956 | ***0.000*** |  |
|  | rs10264272 | T | 0.561 | 1.000 | 1.000 | 1.000 | 1.000 | ***0.000*** | ***0.002*** | ***0.000*** | ***0.000*** | ***0.000*** | ***0.000*** | ***0.000*** | 1.000 | 1.000 | 1.000 | 1.000 | 1.000 | 0.557 | 0.069 | 0.285 | ***0.001*** |  |
|  | rs41303343 | insA | 1.000 | 1.000 | 1.000 | 1.000 | 1.000 | ***0.000*** | ***0.000*** | ***0.000*** | ***0.000*** | ***0.000*** | ***0.000*** | ***0.000*** | 1.000 | 1.000 | 1.000 | 1.000 | 1.000 | 1.000 | 1.000 | 1.000 | 1.000 |  |
| ***CEP72*** | rs924607 | T | 0.116 | 0.427 | ***0.035*** | 0.700 | 0.569 | ***0.000*** | ***0.000*** | ***0.000*** | ***0.000*** | ***0.000*** | ***0.000*** | ***0.000*** | ***0.001*** | 0.339 | 0.105 | ***0.035*** | ***0.011*** | ***0.035*** | 0.115 | 0.068 | 0.329 |  |
| ***ASNS*** | rs3832526 | 3R | ***0.002*** | ***0.035*** | ***0.001*** | ***0.000*** | ***0.017*** | 0.658 | 0.562 | 0.240 | 0.059 | 0.401 | 0.365 | 0.726 | ***0.017*** | 0.317 | 0.213 | 0.321 | ***0.000*** | 0.438 | 0.114 | ***0.000*** | 0.864 |  |
|  | rs1049674 | A | ***0.000*** | 0.051 | ***0.005*** | ***0.004*** | ***0.000*** | ***0.000*** | ***0.000*** | ***0.000*** | ***0.000*** | ***0.000*** | ***0.000*** | ***0.000*** | ***0.001*** | ***0.021*** | ***0.000*** | ***0.000*** | ***0.000*** | ***0.041*** | 0.127 | 0.065 | ***0.004*** |  |
| ***GRIA1*** | rs4958351 | A | 0.982 | ***0.001*** | 0.316 | 0.184 | 0.117 | 0.472 | 0.062 | 0.061 | 0.438 | 0.292 | ***0.015*** | 0.811 | ***0.000*** | ***0.000*** | ***0.000*** | ***0.000*** | ***0.000*** | 0.910 | 0.363 | ***0.000*** | 0.555 |  |
|  | rs11951398 | T | 1.000 | 0.064 | 0.352 | 0.838 | 0.658 | ***0.005*** | ***0.000*** | ***0.015*** | ***0.049*** | ***0.000*** | ***0.005*** | ***0.001*** | ***0.000*** | ***0.000*** | ***0.000*** | ***0.000*** | ***0.000*** | 0.141 | ***0.025*** | ***0.008*** | 0.666 |  |
| *Differenciation test | | | | | | | | | | | | | | | | | | | | | | | | |
