# Supplementary material for: Pharmacogenetics of pediatric acute lymphoblastic leukemia in Uruguay: adverse events related to induction phase drugs
Source: Front Pharmacol. 2023 Nov 17;14:1278769. doi: 10.3389/fphar.2023.1278769 (PMC10690766; doi:10.3389/fphar.2023.1278769)
Supplement: Supplementary file 8 [file Table4.DOCX]

# Supplementary Table 4. Genetic variants and toxicities

| **Genes & Variant** | **Genotype** |  | **Mucositis** | | |  | **Cushing** | | |  | **L-ASP Allergy** | | |  | **Neurotoxicity** | | |
| --- | --- | --- | --- | --- | --- | --- | --- | --- | --- | --- | --- | --- | --- | --- | --- | --- | --- |
|  |  |  | Absence | Presence | p-value* |  | Absence | Presence | p-value* |  | Absence | Presence | p-value* |  | Absence | Presence | p-value* |
| ***ABCB1*** |  |  |  |  |  |  |  |  |  |  |  |  |  |  |  |  |  |
| rs2032582 | CC |  | 41 | 12 | 0.757 |  | 37 | 16 | ***0.033*** |  | --- | --- | --- |  | 47 | 6 | 0.286 |
|  | CA |  | 51 | 18 |  |  | 54 | 15 |  |  | --- | --- |  |  | 56 | 13 |  |
|  | CT |  | 3 | 0 |  |  | 0 | 3 |  |  | --- | --- |  |  | 3 | 0 |  |
|  | AA |  | 0 | 4 |  |  | 11 | 6 |  |  | --- | --- |  |  | 16 | 1 |  |
|  | AT |  | 1 | 1 |  |  | 2 | 0 |  |  | --- | --- |  |  | 1 | 1 |  |
|  | N.A. |  | 44 | 9 | N.A. |  | 29 | 11 | N.A. |  | --- | --- |  |  | 32 | 8 | N.A. |
|  |  |  |  |  |  |  |  |  |  |  |  |  |  |  |  |  |  |
| rs9282564 | TT |  | 108 | 33 | 0.436 |  | 103 | 38 | 0.953 |  | --- | --- | --- |  | 124 | 17 | ***0.005*** |
|  | TC |  | 13 | 6 |  |  | 14 | 5 |  |  | --- | --- |  |  | 12 | 7 |  |
|  | N.A. |  | 19 | 5 | N.A. |  | 16 | 8 | N.A. |  | --- | --- |  |  | 19 | 5 | N.A. |
| ***CYP3A5*** | Expressors^1^ |  | 25 | 2 |  |  | 22 | 5 |  |  | --- | --- | --- |  | 27 | 1 |  |
| rs776746 (*3) |  |  |  |  | ***0.033*** |  |  |  | 0.267 |  |  |  |  |  |  |  | 0.066 |
| rs10264272 (*6) | Non-expressors^2^ |  | 86 | 31 |  |  | 83 | 34 |  |  | --- | --- | --- |  | 96 | 20 |  |
| rs41303343 (*7) | N.A. |  | 29 | 11 | N.A. |  | 28 | 12 | N.A. |  | --- | --- |  |  | 32 | 8 | N.A. |
| ***CEP72*** |  |  |  |  |  |  |  |  |  |  |  |  |  |  |  |  |  |
| rs924607 | CC |  | --- | --- | --- |  | --- | --- | --- |  | --- | --- | --- |  | 50 | 6 | 0.159 |
|  | CT |  | --- | --- |  |  | --- | --- |  |  | --- | --- |  |  | 50 | 13 |  |
|  | TT |  | --- | --- |  |  | --- | --- |  |  | --- | --- |  |  | 25 | 2 |  |
|  | N.A. |  | --- | --- |  |  | --- | --- |  |  | --- | --- |  |  | 30 | 8 | N.A. |
| ***ASNS*** |  |  |  |  |  |  |  |  |  |  |  |  |  |  |  |  |  |
| rs3832526 | 2R2R |  | 73 | 17 | ***0.004*** |  | 61 | 29 | 0.241 |  | 59 | 30 | 0.380 |  | --- | --- | ***---*** |
|  | 2R3R |  | 37 | 11 |  |  | 39 | 9 |  |  | 27 | 22 |  |  | --- | --- |  |
|  | 3R3R |  | 4 | 7 |  |  | 8 | 3 |  |  | 6 | 5 |  |  | --- | --- |  |
|  | N.A. |  | 26 | 9 | N.A. |  | 25 | 10 | N.A. |  | 18 | 17 | N.A. |  | --- | --- |  |
|  |  |  |  |  |  |  |  |  |  |  |  |  |  |  |  |  |  |
| rs1049674 | TT |  | 68 | 24 | 0.352 |  | 73 | 19 | ***0.011*** |  | 54 | 37 | 0.344 |  | --- | --- | ***---*** |
|  | TA |  | 42 | 10 |  |  | 31 | 21 |  |  | 35 | 17 |  |  | --- | --- |  |
|  | N.A. |  | 30 | 10 | N.A. |  | 29 | 11 | N.A. |  | 21 | 20 | N.A. |  | --- | --- |  |
| ***GRIA1*** |  |  |  |  |  |  |  |  |  |  |  |  |  |  |  |  |  |
| rs4958351 | GG |  | --- | --- | --- |  | --- | --- | ***---*** |  | 43 | 30 | 0.223 |  | --- | --- | ***---*** |
|  | GA |  | --- | --- |  |  | --- | --- |  |  | 37 | 15 |  |  | --- | --- |  |
|  | AA |  | --- | --- |  |  | --- | --- |  |  | 7 | 7 |  |  | --- | --- |  |
|  | N.A. |  | --- | --- |  |  | --- | --- |  |  | 23 | 22 | N.A. |  | --- | --- |  |
|  |  |  |  |  |  |  |  |  |  |  |  |  |  |  |  |  |  |
| rs11951398 | CC |  | --- | --- | --- |  | --- | --- | ***---*** |  | 77 | 49 | 0.553 |  | --- | --- | ***---*** |
|  | CT |  | --- | --- |  |  | --- | --- |  |  | 11 | 5 |  |  | --- | --- |  |
|  | N.A. |  | --- | --- |  |  | --- | --- |  |  | 22 | 20 | N.A. |  | --- | --- |  |
| * χ^2^ Test. ^1^ *1/*1 and *1/*3. ^2^ *3/*3, *3/*6 and *3/*7. N.A.: Not available | | | | | | | | | | | | | | | | | |
