# Supplementary material for: Pharmacogenetics of pediatric acute lymphoblastic leukemia in Uruguay: adverse events related to induction phase drugs
Source: Front Pharmacol. 2023 Nov 17;14:1278769. doi: 10.3389/fphar.2023.1278769 (PMC10690766; doi:10.3389/fphar.2023.1278769)
Supplement: Supplementary file 9 [file Table6.DOCX]

# Supplementary Table 6: Ancestry and toxicities

| **Ancestry *** | **Toxicities** | | **p-value**** |
| --- | --- | --- | --- |
|  | **Absence** | **Presence** |  |
|  | **Mucositis** | |  |
| European | 69.5 ± 14.1 | 68.4 ± 12.3 | 0.542 |
| Native American | 20.3 ± 12.6 | 20.8 ± 11.1 | 0.554 |
| African | 10.3 ± 7.4 | 10.8 ± 6 | 0.287 |
|  | **Cushing** | |  |
| European | 68.3 ± 14.1 | 71.7 ± 12.1 | 0.162 |
| Native American | 21.2 ± 12.9 | 18.2 ± 10.1 | 0.273 |
| African | 10.5 ± 7.1 | 10 ± 6.9 | 0.502 |
|  | **L-ASP Allergy** | |  |
| European | 68.2 ± 13.8 | 70.4 ± 13.4 | 0.327 |
| Native American | 21.5 ± 12.0 | 18.6 ± 12.6 | 0.072 |
| African | 10.3 ± 6.9 | 10.9 ± 7.4 | 0.738 |
|  | **Neurotoxicity** | |  |
| European | 68.7 ± 14.0 | 72.6 ± 11.9 | 0.156 |
| Native American | 21.4 ± 12.2 | 14.6 ± 10.9 | ***0.002*** |
| African | 9.9 ± 6.7 | 12.8 ± 8.6 | 0.065 |
| *: Mean ± S.D. (%). **: Mann-Whitney test | | |  |
